# A Study on The Driving Factors and Spatial Spillover of Carbon Emission Intensity in The Yangtze River Economic Belt under Double Control Action

**DOI:** 10.3390/ijerph16224452

**Published:** 2019-11-13

**Authors:** Xuhui Ding, Zhongyao Cai, Qianqian Xiao, Suhui Gao

**Affiliations:** 1 School of Finance and Economics, Jiangsu University, Zhenjiang 212013, China; 2 Institute of Industrial Economics, Jiangsu University, Zhenjiang 212013, China; 3 School of Business Administration, Hohai University, Changzhou 213022, China; caizy99@163.com (Z.C.); xqqhenggaoxing@163.com (Q.X.); 4 China-UK Low Carbon College, Shanghai Jiao Tong University, Shanghai 201306, China

**Keywords:** carbon emission intensity, spatial spillover effect, Yangtze River Economic Belt, double control action

## Abstract

It is greatly important to promote low-carbon green transformations in China, for implementing the emission reduction commitments and global climate governance. However, understanding the spatial spillover effects of carbon emissions will help the government achieve this goal. This paper selects the carbon-emission intensity panel data of 11 provinces in the Yangtze River Economic Belt from 2004 to 2016. Then, this paper uses the Global Moran’s I to explore the spatial distribution characteristics and spatial correlation of carbon emission intensity. Furthermore, this paper constructs a spatial econometric model to empirically test the driving path and spillover effects of relevant factors. The results show that there is a significant positive correlation with the provincial carbon intensity in the Yangtze River Economic Belt, but this trend is weakening. The provinces of Jiangsu, Zhejiang, and Shanghai are High–High agglomerations, while the provinces of Yunnan and Guizhou are Low–Low agglomerations. Economic development, technological innovation, and foreign direct investion (FDI) have positive effects on the reduction of carbon emissions, while industrialization has a negative effect on it. There is also a significant positive spatial spillover effect of the industrialization level and technological innovation level. The spatial spillover effects of FDI and economic development on carbon emission intensity fail to pass a significance test. Therefore, it is necessary to promote cross-regional low-carbon development, accelerate the R&D of energy-saving and emission-reduction technologies, actively enhance the transformation and upgrade industrial structures, and optimize the opening up of the region and the patterns of industrial transfer.

## 1. Introduction

With the frequent occurrence of global warming and haze, low-carbon development has become an important issue in academic areas at home and abroad, which is also an essential requirement for achieving sustainable development and building a community for a common future. According to the estimate of the Carbon Brief, the total amount of carbon emissions in China reached 10 billion tons in 2018. During the same period, the total amount of carbon emissions in the United States and EU was 5.4 billion tons and 3.5 billion tons, respectively. At the 2015 World Climate Conference in Paris, the Chinese government clearly proposed that “carbon emissions will peak around 2030 and will strive to reach the peak as early as possible” and promised that “by 2030, the carbon emissions per unit of GDP will fall by 60–65% compared with 2005” [1]. As the most influential inland economic belt in China, the Yangtze River Economic Belt relies on the Yangtze River Basin and covers 11 provinces. It also connects the Yangtze River Delta urban agglomerations, the urban agglomerations in the middle reaches of the Yangtze River, and the Chengdu–Chongqing urban agglomeration. As an economic demonstration zone for economic transformation and green development, it is especially important to control the total amount and intensity of carbon emissions [2]. The Yangtze River Economic Belt accounts for about 40% of the total carbon emissions with 21.36% of the country’s land area. In the past 20 years, its ecosystem pattern has undergone dramatic changes and its urbanization area has increased by 39.03%. Exploring the trend and inter-regional differences of carbon emissions in the Yangtze River Economic Belt and examining the spillover effects of carbon emissions and its driving factors are particularly important for promoting the low-carbon development of the whole economic belt.

As the global energy crisis and environmental pollution problems have become more pertinent, academic studies on carbon emissions and energy efficiency have become increasingly deep. As early as 1993, Kaya et al. used the ratio of GDP to total carbon emissions to define the concept of carbon productivity [3]. Mielink (1999) brought energy consumption into carbon emission performance assessment, and the carbonization index refers to the total amount of carbon dioxide produced energy consumption per unit. Sun (2005) proposed to measure carbon emission performance by the total amount of carbon emissions generated per unit of GDP, namely, the intensity of carbon emissions [4,5]. Some experts used data envelopment or stochastic frontier methods to measure carbon emission performance and applied the index decomposition method, the input–output method, and the measurement model method to examine the driving factors of carbon emission [6,7,8]. Of course, compared with carbon emission intensity, it is better to use comprehensive indicators or input–output analysis for carbon efficiency, but it is difficult to ensure the accuracy in index and model selection in such studies. Carbon emission intensity or the carbon dioxide emissions per unit of GDP have also been selected to measure the intuitive relationship between economic development and energy utilization [9,10]. Grossman and Krueger (1991) put forward the Environmental Kuznets Curve to explain the relationship between economic growth and environmental pollution. While it is easy to identify the impact of economic growth or per capita income on carbon emissions, this model omits the impact of energy structure, industrial structure, environmental policy, FDI, and other factors [11,12,13]. The threshold regression model, deletion model, and other historical data were commonly used for empirical tests [14]. Many studies have proven that environmental regulation did not always play a positive role in controlling carbon emissions. The cost of regulation compliance will squeeze the investment of technological innovation, and environmental regulations will also lead to the transfer of industry and FDI, which will lead to a pollution haven [15,16].

Considering the natural flow of carbon emissions, the spatial correlation and spillover effects of carbon emissions should not be neglected in the study of regional carbon emissions due to environmental regulation, industrial transfer, and international trade [17,18]. Early scholars mainly used GREEN, GTAP, and other related models to measure inter-regional carbon transfer [19]. Some experts and scholars used input–output tables to analyze the law of inter-regional carbon emission transfers in China [20]. Carbon emissions have significant externalities and spatial spillover effects both globally and within the economic belt. The spatial dependence and spillover effects of environmental governance have attracted academic attention [21,22]. Moreover, some experts have proposed improved multiregional methods to analyze emission spillover and feedback effects among the eight regions or provinces in China [23]. In addition, some experts empirically evaluated the key determinants of carbon emissions at the city-level based on Chinese remote sensing data via spatial autocorrelation [24]. There is a practical need to integrate spatial factors into carbon emission spillover. The spatial Durbin model can better analyze the interaction of environmental strategies and spatial spillover effects induced by local competition [25], which has become the theoretical basis for horizontal ecological compensation. Technological innovation, FDI, environmental regulation, industrial agglomeration, and economic development stages have also been introduced into the spatial econometric model to measure the direction and intensity of the driving factors of carbon emissions in adjacent regions [26]. In this paper, 11 provinces and municipalities in the Yangtze River Economic Belt have been selected as research areas. This paper tightly combines industrial structure evolution and inter-regional transfer with the carbon emissions of the Yangtze River Economic Belt alongside the national politics of Double Control Action into an empirical analysis. With the Moran’s I and spatial econometric models, the spatial correlation of carbon emissions of the Yangtze River Economic Belt and the spatial spillover effect of their driving factors are discussed and have strong theoretical and practical significance.

## 2. Model Construction

### 2.1. Global Moran’s I

The inter-regional transfer trend of industries with high energy consumption and high emissions in the Yangtze River Economic Belt is prominent, and the emissions of CO_2_ and pollutants are obvious cross-regional flow problems, which lead to significant spatial effects of carbon emissions. In addition, some empirical studies have confirmed that the intensity of carbon emissions varied greatly in different regions at different development stages, and there are significant spatial correlations and spatial agglomerations of carbon emissions [27]. To judge whether there is a spatial correlation and heterogeneity among the carbon emissions in the Yangtze River Economic Belt, the Global Moran’s I, which describes spatial autocorrelation, is generally tested. This is also the first question to be answered by using the spatial econometric model to test the spillover effect. The concrete expression of the Global Moran’s I can be seen in Formula (1).

(1) $$I=\sum_{i=1}^{n}\sum_{j=1}^{n}W_{ij}(Y_{i}-\bar{Y})(Y_{j}-\bar{Y})/\sum_{i=1}^{n}\left(Y_{i}-\bar{Y})(Y_{i}-\bar{Y}\right)$$

(2) $$Z=(I-E(I))/\sqrt{Var(I)}$$

where $Y_{i}$ represents the intensity of carbon emissions in region i, $Y_{j}$ represents the intensity of carbon emissions in a specific region j, and n is the number of research objects. $\bar{Y}$ is the average level of carbon emissions for the 11 provinces in the Yangtze River Economic Belt, and $W_{ij}$ is the spatial weight matrix of each province. In Formula (2), Z is a standard normal statistic constructed by Global Moran’s I. When the Z value is significant and positive, there is a positive spatial auto-correlation of carbon emission intensity. In other words, a similar carbon emission intensity trends toward spatial agglomeration. When the Z value is significant and negative, there is a negative spatial auto-correlation of carbon emission intensity, so a similar carbon emission intensity is spatially dispersed. When the Z value is zero, carbon emission intensity is randomly spatially distribution [28]. The paper also discusses the Local Indicators of Spatial Association with a Moran scatter plot, which is used to measure spatial difference and its significance between one region and its surroundings [29]. Of course, a local indicator can also be tested using a LISA figure, which will not be listed for the spatial model. These are all exploratory spatial data analyses that also introduce spatial geography factors.

### 2.2. Spatial Econometric Model

Spatial economics theory holds that spatial decision-making units are affected by the attribute values of units in adjacent areas. The spatial model introduces the geography factor into the estimation with the space matrix W, thereby explaining how the explanatory variables affect the explained variable of the adjacent regions. This model, which can better describe the spatial characteristics and economic phenomena of data, is particularly important [30] since it will explain the possible spatial lag, spatial error, and spatial Durbin model. Evidently, it is necessary to verify spatial correlation through Moran’s I. In addition, if the LM test rejects non-spatial models and accepts both the spatial lag and spatial error models, Lesage and Pace recommended that the spatial Durbin model could be considered to extend the spatial lag model to a Durbin model with spatial lag explanatory variables [31]. In carbon intensity research, carbon dioxide or other pollutants exist within the natural mobility between different provinces. On the other hand, the inter-regional transfer trend of carbon emissions is also been magnified by industrial transfer, domestic trade, and other social factors, which have been proven by many experts [32].

(3) $$y_{it}=\rho {W^{\prime }}_{i}y_{t}+{X^{\prime }}_{it}\beta +\mu_{i}(optional)+\xi_{t}(optional)+\varepsilon_{it}$$

(4) $$y_{it}={X^{\prime }}_{it}\beta +\mu_{i}(optional)+\xi_{t}(optional)+\varphi_{it}\;\varphi i_{t}=\lambda \sum_{i=1}^{N}W_{ij}\varphi i_{j}+\varepsilon_{ij}$$

(5) $$y_{it}=\rho \sum W_{ij}y_{it}+{X^{\prime }}_{it}\beta +\rho \sum W_{ij}Xi_{jt}+\mu_{i}(optional)+\xi_{t}(optional)+\varepsilon_{it}$$

In Formula (3), $y_{ti}$ and $X_{it}$ are the explained variables and explanatory variables of observation unit i at time t; $W_{ij}$ is the preset N∗N order spatial matrix of provinces and municipalities; λ is the self-correlation coefficient of spatial perturbation term; $\mu_{i}$ and $\xi_{i}$ are the spatial and temporal effects respectively; and optional is a selective time and space effect for specific model. Formula (4) is a spatial error model, while Formula (5) is a spatial Durbin model. This model can be used to verify $H_{0}:\theta =0$ and $H_{0}:\theta =\theta +\rho \beta =0$. The first hypothesis tests whether the spatial Durbin model can be simplified to a spatial lag model, and the second hypothesis tests whether it can be simplified to a spatial error model. If both original hypotheses are rejected, the spatial Durbin model can better describe the spatial spillover effect of inter-provincial carbon emissions [33]. Moreover, this paper fully considers the factors of carbon intensity, based on the geospatial carrier of the Yangtze River Economic Belt. While examining the direct influence of explanatory variables, this paper also examines the spatial spillover effects of explanatory variables through spatial conduction mechanisms [34].

## 3. Empirical Test

### 3.1. Spatial Layout of Carbon Emissions

**Figure 1:**
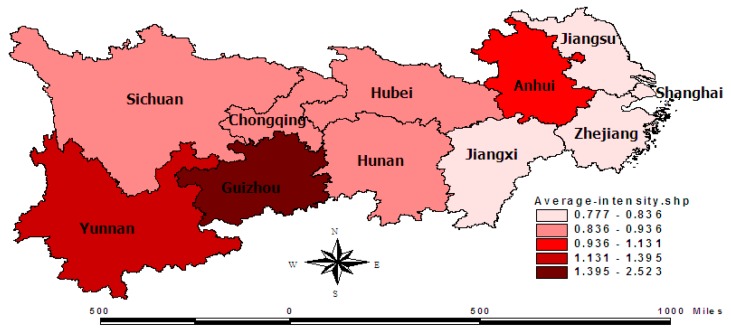
The graduated color figures of average carbon emission intensity from 2004 to 2016.

**Figure 2:**
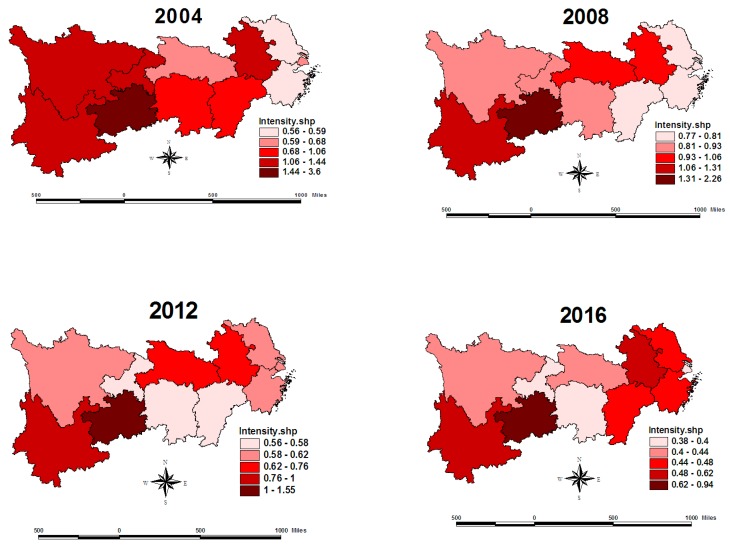
The graduated color figures of carbon emission intensities in 2004, 2008, 2012, and 2016.

Carbon emission intensity is the ratio of the total carbon emissions to regional GDP. Since local provincial governments have not published data for carbon emissions, the standard formula issued by the Intergovernmental Panel on Climate Change (IPCC) is referred in the carbon emission calculation. The measured energy here includes raw coal, coke, crude oil, gasoline, kerosene, diesel, fuel oil, natural gas, and electricity; these data come from the China Statistical Yearbook and China Energy Statistics Yearbook from 2005 to 2017. Because the economic gross of each province has apparent differences, this paper selects the index of carbon emission intensity instead of total carbon emissions. In order to better illustrate the spatial relationship of carbon emission intensity, graduated color figures (Figure 1 and Figure 2) have been adopted with the GIS software.The graduated color figure of the average carbon emission intensity is presented in Figure 1. The four maps in Figure 2 are separately for the years of 2004, 2008, 2012, and 2016. In Figure 1, it can be seen that the color of the Yangtze river delta is lighter, with the lowest level of carbon emission intensity. On the other hand, the color of the Yunnan–Guizhou plateau is heavier, with the highest level of carbon emission intensity. In terms of spatial distribution, the provinces with similar emission intensities are gathered together. Therefore, it been judged that this distribution may have a spatial correlation.

In Figure 2, due to the limited space, only the graduated color figures of some key years are included. Firstly, the overall intensity of carbon emissions declined greatly from 2004 to 2016. For example, the carbon emission per thousand-yuan GDP in Shanghai declined from 0.63 to 0.39 tons, while the carbon emission intensity of Guizhou reduced by about 2.65 tons. Secondly, the regional differences in carbon emission intensities narrowed. However, the ratio of the highest province to the lowest province also fell from 6.06 to 2.46, while the difference between the highest province and lowest province fell from 3.03 to 0.55 tons. Thirdly, the provinces with high emission intensities are gathered together, and the provinces with low emission intensities are gathered together, though the provincial ranking of carbon emission intensity changed during these years. Although there are exceptions for individual provinces in individual years, we could still determine these spatial clustering trends in Figure 2. This is just an intuitive judgment, which will be tested empirically with the universal spatial correlation test. However, the carbon emission intensity of Anhui is higher than the intensity of the surrounding provinces, especially from 2004 and 2016.

### 3.2. Global Spatial Correlation Test

In the test of global spatial correlation, carbon emission intensity was selected as the core indicator to measure carbon emission performance. The Global Moran’s I of the carbon emission intensity of the 11 provinces in the Yangtze River Economic Belt was measured by the Stata and Geoda software. The Durbin model was estimated by Stata, which was also estimated by MATLAB in many previous studies [35]. The specific results are shown in Table 1 and Table 2. In Table 1, the value of Moran’s I was used to measure the aggregation of the carbon emission intensity in the Yangtze River Economic Belt. The spatial weight matrix will select a 0–1 matrix, so the value is 1 when these two provinces are adjacent, and the value is 0 when these two provinces are not adjacent. The Z value is the statistical test value, which uses the theory of standard normal distribution to infer the probability of differences [36]. Only if the Moran’s I passes the test are the spatial models able to estimate the driving factors of carbon emissions. When Moran’ I is greater than 1.96, the spatial correlation should be significant at a 0.05 level.

However, there is no way to determine the specific spatial structure under Moran’s I. Thus, the Local Moran Index was introduced to accurately reflect the agglomeration relationship of carbon emission intensity with the scatter plot [37]. Moran scatter plots can also analyze local spatial features, which are not listed in the text. This paper only lists the final results of the four aggregation types in Table 2. The four quadrants represent four local spatial features, and these features can quantify the spatial correlation between the province and the neighboring provinces in the local region. High–High (H-H) and Low–Low (L-L) types all showed a positive local spatial autocorrelation, while the provinces with similar carbon intensities were gathered together. On the other hand, High–Low (H-L) or Low–High (L-H) types showed spatial heterogeneity, as they are surrounded by different provinces.

### 3.3. Estimation Based on the Spatial Durbin Model

Scholars have paid serious attention to the relationship between economic growth, technological progress, energy structures, and carbon emission intensity, but most empirical studies have adopted a traditional cross-section regression model or panel data model, ignoring the impact of spatial related factors on carbon emissions [38]. In the estimation equation, carbon emission intensity was the explained variable (y in the equation), and the Explanatory variables were selected from the following aspects as x in the equation [39]. The per capita GDP of the region was selected as the variable of economic development, and economic growth contributes to energy conservation and emission reduction through a scale effect, a technical effect, and a structural effect. The proportion of secondary industry to GDP was selected as the variable for industrial structure, and the adjustment of high-emission industries effectively reduces carbon emissions. The number of authorized patents per ten thousand was selected to indicate technological innovation, and production technology advancement and environmental protection technology upgrades can effectively restrain carbon emissions.

The initial stage of urbanization may lead to a high carbonization of people’s lifestyles, and in later stage of urbanization, carbon emissions can also be reduced through a scale effect and an agglomeration effect. The total import and export trade accounts for the proportion of GDP in opening up, and carbon transfer caused by foreign trade directly affects carbon emission intensity. The proportion of foreign enterprise investment in GDP was adopted to show the variable of FDI, while the pollution halo or haven effects need to be tested further. The energy structure refers to the proportion of coal consumption, and the proportion of clean energy directly affects regional carbon intensity [40]. The data of PerGDP, industry structure, urbanization, and openness were taken from the China Statistical Yearbook (2005–2017), the technological innovation data were taken from the China Science and Technology Statistical Yearbook (2005–2017), and the energy structure data were taken from the China Energy Statistics Yearbook (2005–2017). The total carbon emissions of each province were calculated by the standard formula published by the Special Committee on Climate Change (IPCC), because Officials have not released data on carbon dioxide emissions. The FDI data were taken from Provincial Statistical Bulletins. The estimation results can be seen in Table 3.

In selecting the spatial error model, spatial lag model, or spatial Durbin model, LR, LM, and Wald must be tested one by one. Limited by the authors’ knowledge and technical means, the average values from 2004 to 2016 were selected to test. The SEM model is more suitable for spatial models of carbon emissions than the SAR model. The significance level of the LR_spatial_error test value was 0.0082. In other words, the spatial Durbin model cannot be reduced to a spatial error model. However, the *p* value tested by Hausmann is 0.0238, and the null hypothesis that “individual variables do not change with time” should be rejected. The fixed effect model should be adopted, and the time effect should be considered in the model. The spatial Durbin model fixing time and space were adopted [41]. In addition, some spatial cross terms of the independent variables did not pass the significance test. In order to optimize the results of the independent variable regression, only the spatial spillover effects of economic development, industrial structure, technological innovation, and other non-spatial driving factors in the formation of carbon emission intensity were considered. Of course, many experts also merely adopted non-spatial models to estimate the driving factors of carbon emissions, such as exponential decomposition, the stochastic frontier analysis generalized method of moments, and the sample selection model [42,43].

## 4. Discussion of Results

### 4.1. Discussion of Global Spatial Correlation

According to the estimation results in Table 1, the Moran’s I of carbon intensity for the Yangtze River Economic Belt from 2004 to 2006 is positive, and the Z test value is greater than 1.96., which passes the significance test. This indicates that carbon emission intensity has a significant spatially positive correlation, so the spatial model is necessary to explore why the spatial effects appear. Provinces with a higher carbon emission intensity or lower carbon emission intensity have significant spatial agglomeration, which has significant characteristics of the “Matthew effect”. Changes in the carbon emission intensity are strongly influenced by spatial correlation factors. Neglecting spatial factors will lead to a deviation between the model estimation and empirical conclusions [44]. In most past studies, the spatial spillover effect of carbon intensity has been ignored, so estimates of the driving factors were not accurate, while the Moran Index and Z value passed the significance test in Table 1. From the perspective of development process, the spatial correlation of carbon emission intensity in the Yangtze River Economic Belt basically maintains a nonlinear trend of “down-up-down”. Spatial autocorrelation showed a downward trend from 2005 to 2008, presented a spiral upward trend from 2008 to 2011, reached the highest spatial correlation in 2011, and showed a sharp downward trend from 2011 to 2016. In the process of economic growth, industrial structures and environmental regulations have undergone major changes among these provinces. Based on the spatial correlation findings in Table 1, spatial models should be constructed to estimate the driving factors.

Table 2 shows the local spatial autocorrelation structure for the spatial agglomeration of carbon emission intensity in the Yangtze River Economic Belt. It can be seen that most provinces are classified into a High–High agglomeration or a Low–Low agglomeration, while only two or three minority provinces are classified into High–Low or Low–High agglomerations. The provinces with a Low–Low agglomeration are mainly concentrated in the Yangtze River Delta. Due to the high level of economic development and strong technological innovation in Jiangsu, Zhejiang, and Shanghai, local governments have introduced strict environmental regulations on energy conservation and emission reduction. In terms of carbon emission trading, Hubei Province has always been the first in the number and capital scale of its carbon financial products, which has produced new momentum for green development [45]. The provinces with High–High agglomeration are mainly concentrated in Yunnan, Guizhou, and other provinces. In pursuit of regional economic growth, these provinces inevitably have relaxed their environmental regulations or engaged in some industries with high energy consumption and emissions. For High–Low and Low–High agglomeration, Anhui Province has become a depression area for carbon emission control in the Yangtze River Delta due to its lagging economic development and poorer technological innovation. However, Sichuan, Chongqing, and other provinces have become strategic forces for economic growth in the western region, which have obvious advantages in attracting high-tech industries and scientific and technological talents, so their carbon emission intensities are lower than those of the surrounding provinces [46].

### 4.2. Discussion of Driving Factors

Based on the non-spatial estimated results in Table 3, economic growth, technological innovation, and FDI have significant negative effects on regional carbon emission intensity. However, industrial structure and industrialization level have significant positive effects on carbon emission intensity. When economic development reaches a certain level, people’s awareness of energy conservation and emission reduction is strengthened. The dependence of economic growth on carbon emissions has gradually weakened, so simple and pollutive growth has begun to change to a low-carbon economy [47]. By the end of 2016, the per capita GDP of most provinces in the Yangtze River Economic Belt, except Yunnan and Guizhou, exceeded 5000 dollars. On the whole, the EKC curve of carbon emissions passed the inflection point of emissions and began to decline [48]. For the Environmental Kuznets Curve, environmental quality decreases at low income levels as per capita GDP increases, while environmental quality rises with GDP growth at high income levels, which is a U-shaped trend [49]. Carbon emissions at a high-income level decreased with the growth of per capita of GDP. Economic growth is not a sufficient condition for environmental improvement or a low-carbon economy, but economic growth is a necessary condition for carbon emission control. In addition, technological innovation is a fundamental way to improve carbon emission efficiency and develop a low-carbon economy. With regards to the economic development, the improvement of environmental quality is also accompanied by technological progress. The alternatives of clean energy sources and technology for carbon capture or carbon storage are inseparable with technological innovation [50]. Some experts, however, have argued that technological innovation would lead to an acceleration of output, which would lead to an increase in energy use and carbon emissions [51].

Whether FDI could reduce or increase the intensity of carbon emissions while promoting economic growth remains controversial in academic circles. The “Pollution Halo Hypothesis” holds that green production technology transmitted by foreign-funded enterprises with advanced technologies would effectively reduce carbon emissions. However, the “Pollution Halo Hypothesis” suggests that developed countries transfer carbon-intensive industries to developing countries [52,53]. Studies have confirmed that FDI has introduced greener low-carbon concepts and energy-saving and emission reduction technologies. In recent years, the Chinese government has also greatly improved its environmental supervision and entry standards for FDI [54]. Economic structural transformation and industrial structure upgrading are important driving factors for suppressing carbon intensity. In industrialized countries, rapid economic growth also means the rapid growth of energy consumption and carbon emissions [55]. Many studies have confirmed that there is a stable relationship between the proportion of the manufacturing industry and carbon emissions. Thus, an increase in carbon emissions per unit output in primary and secondary industries is significantly higher than that in tertiary industries. In the process of industrialization, carbon emission intensity may be an inverted u-shape. The natural evolution of economic structures began with a “clean” agricultural economy, then entered a “polluted” industrial economy, and finally changed into a “cleaner” service economy [56]. Good intentions do not always lead to good results, so carbon intensity may be improved only when industrialization reaches a certain stage and tertiary industry increases [57]. Many scholars have performed significant theoretical research and empirical tests on the green paradox, so policies for the design of energy and climate must consider the specific industrial stage and economic development [58].

### 4.3. Discussion of Spatial Spillover Test

Based on the spatial spillover estimation in Table 3, it can be seen that industrialization has a significant positive effect on the carbon emission intensity of neighboring provinces. In other words, manufacturing agglomeration and industrial structure adjustment had a transferring effect on surrounding areas, thereby affecting the energy consumption and carbon emissions of the surrounding areas [59]. Relying on the golden waterway, the Yangtze River Economic Belt has been building a regional industrial chain. Some energy-intensive and emission-intensive industries are moving to central and western regions. The environmental pollution in the Yangtze River Basin for decades has always been closely related to industrial transfer. The carbon emissions of each province in the Yangtze River Economic Belt do not exist independently in geographical space, while both carbon emissions and manufacturing have strong spatial agglomeration and spatial interaction effects. This conclusion has been confirmed by the Global Moran’s I test results in Table 3 [60]. In addition, due to administrative divisions, the promotion of officials, and local competition, the division and cooperation of the regional industrial chain are increasingly limited in the administrative region. However, the positive effects precipitated by manufacturing agglomeration and industrialization have not spread across the provinces in the Yangtze River Economic Belt [61]. In the process of industrial structure adjustment on the east coast, excess industries have transferred to central and western regions. This process can also be regarded as the transfer of high-carbon emission products from underdeveloped regions to central and western regions through inter-regional trade. There is still a long way to go to achieve low-carbon and green coordinated development in the Yangtze River Economic Belt [62]. Many Experts have verified the transfer of embodied carbon in international trade, and the evolution of industrialization in some developed countries could also lead to some high emission industries moving from developed countries to developing countries [63,64]. This situation also exists in inter-regional industrial transfers and domestic trade, and the construction of the Yangtze River Economic Belt is essentially a process of industrial division and transfer, which occurs in developed countries [65].

As can be seen in Table 3, technological innovation has obvious negative effects on the carbon emission intensity of neighboring provinces with a spatial spillover effect. Technological innovation could decrease the carbon emission level of neighboring regions, which can significantly inhibit the growth of carbon emissions and boost green low-carbon development [66]. Indeed, not only carbon dioxide and other gas emissions have strong spatial mobility; technological innovation also has significant spatial spillover characteristics, which decrease gradually with geographical distance [67]. A large number of recent studies have clearly demonstrated that low-carbon technology innovation also has the characteristics of time–space and spatial spillover. Therefore, related studies must abandon the traditional assumption that the impact process is an innocent closed system and that each factor only affects local hypotheses [68,69]. The spatial spillover of technological innovation will be realized through demonstration, imitation, and information sharing among enterprises, as well as inter-regional trade, exchange and cooperation, talent flow, and so on. The agglomeration effect and diffusion effect of technological innovation are the endogenous driving forces of green coordinated development in the Yangtze Economic Belt [70]. A large amount of research has been accumulated on the innovative attributes of low-carbon technology innovation and climate change mitigation. On the other hand, Su and Moaniba also initially demonstrated that climate change and carbon emissions directly promoted low-carbon technology innovation [71]. In addition, inter-regional industrial transfer plays the dual role of devil and angel in carbon emissions for the receiving regions. However, technological innovation spillover undoubtedly plays a significant role in spreading green and low-carbon technologies, which can partly offset the pollution haven effect [72]. However, the existence of this dual effect makes the spatial spillover effect unclear, as this effect depends on the specific type of inter-regional industrial transfer, the stage of economic development, and the spatial structure of carbon emission intensity, which needs further research.

### 4.4. Discussion of Direct and Indirect Effects

The direct effect and indirect effect of decomposition further reveal the spatial spillover effect of each influencing factor on carbon emission intensity. The direct effect represents how a factor affected the intensity of the local region, while the indirect effect inversely represents how a factor affected the intensity of the adjacent region [73]. As seen in Table 4, the direct and indirect effects also have a significant positive effect, while industrial agglomeration exacerbates carbon emissions in local and adjacent provinces. This also implies that upgrading industrial structures and industrial cooperation is crucial to the green development of the whole Yangtze river economic belt [74]. On the other hand, technology innovation had a negative effect on the carbon emissions of local and adjacent provinces, thereby curbing regional carbon emissions with technological progress in energy conservation and emission reduction [75]. With the rapid development of convenient transportation and Internet technology, advanced technology can be effectively spread and exchanged, in order to promote the development and application of technology in the whole economic belt. The economic level and FDI only have negative effects on carbon emissions; however, their indirect effects on adjacent regions were not significant. The estimation results in Table 3 are exactly in accordance with the estimated results in Table 4, which further verifies the existence of the spatial spillover effect.

## 5. Conclusions

Given the energy crisis and exhaust emissions, it is necessary to explore the spatial structures, driving factors, and spillover effects of carbon emission intensity in the Yangtze River Economic Belt. However, regional collaboration is also an inevitable choice for promoting high-quality green development. This study selected the carbon intensity data of 11 provinces in the Yangtze River Economic Belt from 2004 to 2016 and used the Global Moran’s I and scatter plot to test the spatial correlation characteristics of carbon emission intensity. This study also constructed a spatial Dubin model to empirically estimate the driving factors and spatial spillover effects. The results show that the Moran’s I of carbon intensity in Yangtze River Economic Belt is significantly positive, so there is a significant positive correlation with the provincial carbon emission intensity. In addition, the spatial correlation shows a trend of “down-up-down”. The provinces with Low–Low agglomeration are mainly concentrated in the Yangtze River Delta, and the High–High agglomerations are mainly concentrated in the provinces of Yunnan and Guizhou, while the Sichuan, Chongqing, and Anhui provinces show the spatial heterogeneity of carbon intensity. The estimated results of the driving factors, economic growth, technological innovation, and FDI have significant negative effects on carbon emission intensity. The industrial structure or industrialization level has a positive effect on carbon emission intensity. From the perspective of spatial spillover, industrialization has a significant positive effect on the carbon intensity of neighboring provinces, while technological innovation has a negative effect. The per capita GDP did not pass the significance test for the spatial spillover effect. Therefore, policy design must consider the regional interactions between carbon intensity, its driving factors, and cross-regional carbon control.

The government should promote the coordinated development of low carbon in the Yangtze River Economic Belt from the following aspects. Firstly, the spatial spillover effects of carbon emission intensity should be highly valued, the spatial transmission mechanism of carbon transfer also should be accurately determined, and the reduction targets of carbon emissions should be rationally developed. Secondly, the problems of carbon emissions should also be solved in economic development. In other words, China should comprehensively promote the transformation and upgrading of industrial structures, strictly control and eliminate backward production capacities, and focus on the development of a modern service industry and high-tech industry. Thirdly, the government should strictly implement an environmental access system, restrict FDI flowing to high investment and high pollution industries, guide FDI into the environmental protection field, and unleash a green spillover effect. Fourthly, the government should fully accelerate the R&D of environmental-protection technologies, enhance the technological breakthroughs of clean energy, and realize the exchange and innovation of technological innovation in the Yangtze River Economic Belt. Fifthly, the government should develop strict environmental regulations, change the evaluation standards for the performance of local officials, prohibit the inter-regional transfer of high-emission industries, and build a single economic community based on green development. In the future, the carbon emission intensity in different industries should be considered for the development of different industry policies. On the hand, the international transfer and domestic transfer of carbon emissions must be considered together, and the countries of origin and inflowing industries should be clearly distinguished, in order to formulate precise policies for the Yangtze River Economic Belt.

## Figures and Tables

**Table 1:** Global Moran’s I Measurement Results of Carbon Emission Intensity in the Yangtze River Economic Belt from 2005 to 2016.

Year	Moran’s I	Z Value	Year	Moran’s I	Z Value
2004	0.310	4.583	2011	0.287	3.699
2005	0.319	4.798	2012	0.212	3.087
2006	0.226	4.058	2013	0.164	2.667
2007	0.275	4.412	2014	0.177	2.518
2008	0.260	3.930	2015	0.127	2.049
2009	0.283	3.722	2016	0.122	1.965
2010	0.251	3.325			

Note: A Z value greater than 1.96 is a 0.05 significant level, while 2.58 is a 0.01 significant level.

**Table 2:** Corresponding Provinces of Moran’s I Scatter Point Map of Carbon Emission Intensity in the Yangtze River Economic Belt.

Year	H-H Agglomeration	L-H Agglomeration	L-L Agglomeration	H-L Agglomeration
2005	Chongqing, Guizhou, Yunnan	Hunan, Sichuan	Hubei, Jiangsu, Jiangxi, Shanghai, Zhejiang	Anhui
2009	Guizhou, Yunnan	Chongqing, Hunan, Sichuan	Hubei, Jiangsu, Jiangxi, Shanghai, Zhejiang	Anhui
2011	Guizhou	Chongqing, Hunan, Sichuan, Yunnan	Hubei, Jiangsu, Jiangxi, Shanghai, Zhejiang	Anhui
2016	Guizhou, Yunnan	Chongqing, Hunan, Sichuan	Hubei, Jiangsu, Jiangxi, Shanghai, Zhejiang	Anhui

**Table 3:** Estimation of Carbon Emission Intensity Driven by the Spatial Dubin Model in the Economic Belt of the Yangtze River from 2004 to 2016.

Variable	Coef.	Std. Err.	z	*p* > |z|
PerGDP	−2.405238	0.500307	−4.81	0
industry	2.30355	0.835597	2.76	0.006
tech	−0.0123936	0.005838	2.12	0.034
city	0.961454	1.52161	0.63	0.527
open	−0.0469965	0.200025	−0.23	0.814
FDI	−0.0231999	0.013819	−1.68	0.093
energy	0.526055	0.464765	1.13	0.258
W*PerGDP	0.697681	1.047414	0.67	0.505
W*industry	5.722082	2.536778	2.26	0.024
W*tech	−0.0377139	0.017677	2.13	0.033

R-sq: within = 0.6175; between = 0.2884; overall = 0.3678; Mean of the fixed-effects = 12.6125; Log-likelihood = 45.3741.

**Table 4:** The Direct Effect, Indirect Effect, and Overall Effect of the Spatial Dubin Model from 2004 to 2016.

Variables	Direct Effect	Indirect Effect	Overall Effect
PerGDP	−2.1815 (−5.36)	0.7931 (1.16)	−1.3884 (−1.92)
industry	3.2517 (4.06)	3.1744 (2.02)	6.4261 (3.33)
tech	−0.0187 (3.66)	−0.0209 (1.08)	−0.0397 (3.29)
city	0.8969 (0.61)	−0.1273 (−0.46)	0.7695 (0.61)
open	−0.0380 (−0.20)	0.0056 (0.17)	−0.0323 (−0.20)
FDI	−0.0220 (−1.66)	0.0036 (1.11)	−0.0184 (−1.66)
energy	0.5234 (1.20)	−0.0774 (−0.88)	0.4459 (1.17)

## References

[1] Mo J.L., Duan H.B., Fan Y., Wang S.Y. (2018). China’s Energy and Climate Targets in the Paris Agreement: Integrated Assessment and Policy Options. Econ. Res. J..

[2] Huang G.H., Liu C.J., Xu Z.H. (2018). Carbon Emission Reduction Potential and Low-Carbon Development Strategy in Yangtze River Economic Belt. Resour. Environ. Yangtze Basin.

[3] Kaya Y., Yokobori K. (1998). Environment, Energy and Economic: Strategies for Sustainability.

[4] Mielnik O., Goldemberg J. (1999). The evolution of the “carbonization index” in developing countries. Energy Policy.

[5] Sun J.W. (2005). The Decrease of CO_2_ Emission Intensity is Decarbonization at National and Global Levels. Energy Policy.

[6] Ding X.H., Zhang Z.X., Wu F.P. (2019). Threshold Effect of Environmental Regulation on Regional Carbon Emission Performance under Double Control Action. East China Econ. Manag..

[7] Cansino J.M., Sanchez-Braza A., Rodriguez-Arevalo M.L. (2015). Driving forces of Spain’s CO_2_ emissions: A LMDI decomposition approach. Renew. Sustain. Energy Rev..

[8] Jin T., Kim J. (2019). A Comparative Study of Energy and Carbon Efficiency for Emerging Countries using Panel Stochastic Frontier Analysis. Sci. Rep..

[9] Wang S.J., Huang Y.Q. (2019). Spatial Spillover Effect and Driving Forces of Carbon Emission Intensity at City Level in China. Acta Geogr. Sin..

[10] Han X.Y., Cao T.Y., Sun T. (2019). Analysis on the Variation Rule and Influencing Factors of Energy Consumption Carbon Emission Intensity in China’s Urbanization Construction. J. Clean. Prod..

[11] Grossman G.M., Krueger A.B. (1991). Environmental Impacts of the North American Free Trade Agreement. NBER Dordrecht. Work. Pap..

[12] Wang X.E., Duan Z.Y., Wang P.B., Song J.N., Wang S., Duan H.Y. (2018). Study on measurement of carbon-driving effects from technological change and structural adjustment in typical countries from 1990 to 2014. Resour. Sci..

[13] Wang Y.N., Zuo Y.H., Chen W., Wang B.W. (2018). Threshold Effect and Regional Differences of Environmental Regulation on Carbon Emission. Res. Environ. Sci..

[14] Yang H.R., Zheng H., Liu H.G., Wu Q. (2019). Non-Linear Effects of Environmental Regulation on Eco-Efficiency under the Constraint of Land Use Carbon Emissions: Evidence Based on a Bootstrapping Approach and Panel Threshold Model. Int. J. Environ. Res. Public Health.

[15] Khan Z., Zhu S.S., Yang S.Q. (2017). Environmental Regulations an Option: Asymmetry Effect of Environmental Regulations on Carbon Emissions using Non-linear ARDL. Energy Sour. Part A Recovery Util. Environ. Eff..

[16] Zhang H., Wei X.P. (2014). Green Paradox or Forced Emission reduction: Dual Effect of Environmental Regulation on Carbon Emissions. China Popul. Resour. Environ..

[17] Wiebe K.S. (2018). Identifying Emission Hotspots for Low Carbon Technology Transfers. J. Clean. Prod..

[18] Sun L.C., Cheng F.X., Li Q. (2014). Characteristics and Economic Spillover Effect of the Regional Carbon Emissions Transfer. China Popul. Resour. Environ..

[19] Shi M.J., Wang Y., Zhang Z.Y., Zhou X. (2012). Regional Carbon Footprint and Interregional Transfer of Carbon Emissions in China. Acta Geogr. Sin..

[20] Yao L., Liu J.R. (2010). Transfer of carbon emissions between China’s eight major regions. China Popul. Resour. Environ..

[21] Shen K.R., Jin G., Fang X. (2017). Does Environmental Regulation Cause Pollution to Transfer Nearby?. Econ. Res. J..

[22] Zhao X.W. (2014). Environmental Regulation, Environmental Regulation Competition and Regional Industrial Economic Growth: An Empirical Study Based on Spatial Panel Durbin Model. J. Int. Trade.

[23] Ning Y.D., Miao L., Ding T., Zhang B.Y. (2019). Carbon Emission Spillover and Feedback Effects in China based on a Multiregional Input-output Model. Resour. Conserv. Recycl..

[24] Su W.S., Liu Y.Y., Wang S.J., Zhao Y.B., Sun Y.X., Li S.J. (2018). Regional Inequality, Spatial Spillover Effects, and the Factors Influencing City-level Energy-related Carbon Emissions in China. J. Geogr. Sci..

[25] Fan H., Zhou D.Q. (2012). On the Total Factor Energy Efficiency with Undesirable Outputs in China. J. Appl. Stat. Manag..

[26] Wang Y.P., Li J. (2019). Spatial Spillover Effect of Non-fossil Fuel Power Generation on Carbon Dioxide Emissions across China’s Provinces. Renew. Energy.

[27] Wu Y.M. (2010). Estimation of Input-Output Elasticity of Regional Agricultural Production Elements in China: An Empirical Study Based on Spatial Econometric Model. Chin. Rural Econ..

[28] Hong S.Y., Wang H.R., Lai W.L., Zhu Z.F. (2017). Spatial Analysis and Coordinated Development Decoupling Analysis of Energy-consumption Water in China. J. Nat. Resour..

[29] Hu Y.X., Pan J.H., Wang Y.R. (2015). Spatial-temporal Evolution of Provincial Carbon Emission in China from 1997 to 2012 based on ESDA and GWR Model. Acta Sci. Circumst..

[30] Zhou Y.Y., Xu Y.R., Liu C.Z., Fang Z.Q., Guo J.Y. (2019). Spatial Effects of Technological Progress and Financial Support on China’s Provincial Carbon Emissions. Int. J. Environ. Res. Public Health.

[31] Lesage J.P., Pace R.K. (2009). Introduction to Spatial Econometric.

[32] Li J., Luo Y., Wang S.Y. (2019). Spatial Effects of Economic Performance on the Carbon Intensity of Human Well-being: The Environmental Kuznets Curve in Chinese Provinces. J. Clean. Prod..

[33] Lee L.F., Yu J. (2012). Spatial Panel: Random Components versus Fixed Effects. Int. Econ. Rev..

[34] Xu Y.Z., Chen Y. (2018). Spatial Spillover Effects of Carbon Lock-in among Provinces in China——An Empirical Study Based on Spatial Auto-regressive Model. J. South China Norm. Univ..

[35] Zhou B., Zhang C., Song H.Y., Wang Q.W. (2019). How does Emission Trading Reduce China’s Carbon Intensity? An Exploration using a Decomposition and Difference-in-differences Approach. Sci. Total Environ..

[36] Li C., Feng W., Shao G.L. (2018). Spatio-Temporal Difference of Total Carbon Emission Efficiency of Fishery in China. Econ. Geogr..

[37] Lu X.L. (2014). The Carrying Capacity Evaluation and Spatial Statistical Analysis of Provincial Resource and Environment in China. Stat. Decis..

[38] Zaidi S.A.H., Zafar M.W., Shahbaz M., Hou F.J. (2019). Dynamic Linkages between Globalization, Financial Development and Carbon Emissions: Evidence from Asia Pacific Economic Cooperation Countries. J. Clean. Prod..

[39] Wang F., Wu L.H., Yang C. (2010). Driving Factors for Growth of Carbon Dioxide Emissions during Economic Development in China. Econ. Res. J..

[40] Dong M., Xu Z.Y., Li C.F. (2018). Analysis of Energy-saving and Emission Reduction Effect under the Constraint of Carbon Intensity. Soft Sci.

[41] Lee L.F., Yu J. (2010). A Spatial Dynamic Panel Dada with both Time and Individual Fixed Effects. Econ. Theory.

[42] Wu X.R., Zhang J.B., Tian Y., Li P. (2014). Provincial Agricultural Carbon Emissions in China: Calculation, Performance Change and Influencing Factors. Resour. Sci..

[43] Zhang J.C., Zhong Z.W. (2015). Research of Chinese Provincial Carbon Efficiency and Total Factor Productivity Based on SFA. Soft Sci..

[44] Wang S.J., Huang Y.Y. (2019). Spatial Spillover Effect and Driving Forces of Carbon Emission Intensity at City Level in China. Acta Geogr. Sinica.

[45] Shu X., Xia C.Y., Li Y., Tong J., Shi Z. (2018). Relationships between carbon emission, urban growth, and urban forms of urban agglomeration in the Yangtze River Delta. Acta Ecol. Sin..

[46] Chen J., Cheng D.X., Zhu D.J. (2012). Evaluation of Low Carbon Competitiveness of Chinese Cities Based on Grey Ideal Relevance Analysis. Resour. Sci..

[47] Akalpler E., Hove S. (2019). Carbon Emissions, Energy Use, Real GDP Per Capita and Trade Matrix in the Indian Economy—An ARDL Approach. Energy.

[48] Gill A.R., Viswanathan K.K., Hassan S. (2018). A Test of Environmental Kuznets Curve (EKC) for Carbon Emission and Potential of Renewable Energy to Reduce Green House Gases (GHG) in Malaysia. Environ. Dev. Sustain..

[49] Xu G.Y., Song D.Y. (2010). An Empirical Study of the Environmental Kuznets Curve for China Carbon Emissions—Based on Provincial Panel Data. China Ind. Econ..

[50] Ganda F. (2019). The Impact of Innovation and Technology Investments on Carbon Emissions in Selected Organisation for Economic Co-operation and Development Countries. J. Clean. Prod..

[51] Li B. (2013). District Technological Innovation Capacity and Carbon Emission Per Capita of China. Soft Sci..

[52] Huynh C.M., Hoang H.H. (2019). Foreign Direct Investment and Air Pollution in Asian Countries: Does Institutional Quality Matter?. Appl. Econ. Lett..

[53] Sun H., Clottey S.A., Geng Y., Fang K., Amissah J.C.K. (2019). Trade Openness and Carbon Emission: Evidence from Belt and Road Countries. Sustainability.

[54] Liu M.K., Fu S.Y. (2018). Differential analysis about different sources of FDI on China’s carbon emission: Based on China’s inter-provincial panel data from 1996 to 2016. Sci. Technol. Manag..

[55] Yao C.R. (2015). Study on the Difference of Carbon Emission Drivers between Industrialized Countries and Emerging Market Economy. World Econ. Stud..

[56] Wu L.J., Tian Q.B. (2016). Time Trend and Regional Difference of Carbon Emissions in China: Based on the Evolution Law of Carbon Emissions in the Process of Industrialization. J. Shanxi Univ. Financ. Econ..

[57] Li Y.T. (2015). The Literature Review on Green Paradox of Climate Policy. Mod. Econ. Res..

[58] Wu G.Z., You D.M. (2018). Re-analysis of Green Paradox: Based on the Perspective of Economic Policy Uncertainty. Syst. Eng..

[59] Zhang Y.J., Liu Z., Zhang H., Tan T.D. (2014). The Impact of Economic Growth, Industrial Structure and Urbanization on Carbon Emission Intensity in China. Nat. Hazards.

[60] Liao S.H., Xiao Y.F. (2017). Pollution Industry Transfer and Carbon Transfer Space Characteristic in Midland of China. Econ. Geogr..

[61] Feng W., Ji G.J., Pardalos P.M. (2019). Effects of Government Regulations on Manufacturer’s Behaviors under Carbon Emission Reduction. Environ. Sci. Pollut. Res..

[62] Li F.J. (2018). Research Progress and Prospect of Implicit Carbon Emission Transfer in Interregional Trade. Prog. Geogr..

[63] Dong D., An H.Z., Huang S.P. (2017). The Transfer of Embodied Carbon in Copper International Trade: An Industry Chain Perspective. Resour. Policy.

[64] Mongelli I., Tassielli G., Notarnicola B. (2006). Global Warming Agreements, International Trade and Energy/Carbon Embodiments: An Input-output Approach to the Italian Case. Energy Policy.

[65] Du P.L., Wang A.G. (2018). The Economic Landscape of the World Carbon Transfer and China’s Midstream Status: An Empirical Analysis based on Network Governance. World Econ. Stud..

[66] Li L., Hong X.F., Wang J. (2018). Spatial Effects of Technology Innovation on Energy-Related Carbon Emission: A Spatial Panel Approach. Fresenius Environ. Bull..

[67] Wang W.D., Lu N., Zhang C.J. (2018). Low-carbon technology innovation responding to climate change based on perspective of spatial spillover effect. China Popul. Resour. Environ..

[68] Su H.N., Moaniba I.M. (2017). Does Innovation Respond to Climate Change? Empirical Evidence from Patents and Greenhouse Gas Emissions. Technol. Forecast. Soc. Chang..

[69] Anselin L. (2001). Spatial Effects in Econometric Practice in Environmental and Resource Economics. Am. J. Agric. Econ..

[70] Chen W.D., Yang R.Y. (2018). Evolving Temporal-Spatial Trends, Spatial Association, and Influencing Factors of Carbon Emissions in Mainland China: Empirical Analysis Based on Provincial Panel Data from 2006 to 2015. Sustainability.

[71] Sun H., Bless K.E., Sun C., Kporsu A.K. (2019). Institutional Quality, Green Innovation and Energy Efficiency. Energy Policy.

[72] Xu J., Zhou M., Xia Q. (2017). Dynamic Effects and Influencing Mechanisms of Carbon Emissions from Interprovincial Industrial Regional Transfer in China. J. China Univ. Geosci. Soc. Sci. Ed..

[73] Zhang C.J., Bai Q., Zhang W.A. (2017). The Influencing Factors of Regional Carbon Emission Intensity in China and Spatial Spillover. Syst. Eng..

[74] Ding X.H., Tang N., He J.H. (2019). The Threshold Effect of Environmental Regulation, FDI Agglomeration and Water Use Efficiency under “Double Control Actions”—An Empirical Test Based on Yangtze River Economic Belt. Water.

[75] Liu Z.K., Li X. (2019). Has China’s Belt and Road Initiative Promoted its Green Total Factor Productivity?. Energy Policy.

